# A Six-Month Observational Study of Nursing Workload in 14 Latvian Intensive Care Units Using the Nursing Activities Score

**DOI:** 10.3390/healthcare14010134

**Published:** 2026-01-05

**Authors:** Olga Cerela-Boltunova, Inga Millere

**Affiliations:** Department of Nursing and Midwifery, Riga Stradiņš University, LV-1067 Riga, Latvia

**Keywords:** intensive care units, nursing workload, personnel staffing and scheduling, health workforce, Latvia

## Abstract

**Highlights:**

**What are the main findings?**

- Nursing workload in Latvian ICUs is consistently high, with a mean Nursing Activities Score (NAS) of 65.45 points, corresponding to approximately 15.7 h of nursing care per patient per day.
- Significant interunit and interlevel variability was identified, with nursing shortages strongly associated with NAS-based workload rather than shift type or ICU level.

**What are the implications of the main findings?**

- Fixed nurse-to-patient ratios are insufficient to reflect real care demands; NAS-based workload measurement should be integrated into routine staffing and workforce planning.
- Objective workload data provide a robust evidence base for national ICU staffing reforms aimed at improving patient safety and nurse well-being.

**Abstract:**

**Objectives:** Intensive care units (ICUs) are characterised by high care complexity and nursing workload, which directly affects patient safety and staff sustainability. Latvia faces a chronic shortage of nurses, particularly in intensive care, yet systematic national data on nursing workload have been lacking. This study aimed to quantitatively assess nursing workload in Latvian ICUs using the Nursing Activities Score (NAS) and to evaluate its relationship with staffing adequacy. **Methods:** A prospective, multicentre observational study was conducted over six months (May–November 2025) in 14 Latvian ICUs representing all three levels of intensive care. Nursing workload was measured using the NAS during each 12 h shift. A total of 28,079 complete NAS observations were analysed using descriptive statistics, inferential tests (*t*-tests, ANOVA), mixed-effects modelling, regression analysis, and time-series forecasting. **Results:** The mean NAS was 65.45 (SD = 25.76), equivalent to an average of 15.71 nursing care hours per patient per day. Workload remained similarly high during day and night shifts. Significant differences were observed between ICUs and care levels, with level 2 units showing the highest workload. The average nursing shortage rate was 42.6% and was strongly predicted by NAS values (R^2^ = 0.115), whereas shift type and unit level had minimal explanatory power. **Conclusions:** ICU nursing workload in Latvia is persistently high and unevenly distributed across units. Staffing levels are not adequately adjusted to actual care demands. Integrating NAS-based workload monitoring into staffing models is essential for evidence-based workforce planning, improving patient safety, and reducing nurse overburdening.

## 1. Introduction

ICUs (intensive care unit) are the healthcare environment with the highest patient care intensity, where sufficient nursing staff is critical for ensuring high-quality and safe care [1]. The matter of nursing workload and staff shortages in ICUs has been raised in many countries in recent years and was particularly exacerbated by the COVID-19 pandemic [2]. These challenges are striking in Latvia because the number of nurses per capita is significantly lower than the EU average—around 4.2 nurses per 1000 inhabitants, which is less than half the EU average of ~8.5, and the healthcare system faces a persistent shortage of qualified staff [3]. In particular, there is an acute shortage of nurses in ICUs in Latvia: out of almost 9000 active nurses, only around 600 specialise in intensive care and are registered nurses with a specialisation, and due to limited resources, one ICU nurse often has 2–3 critically ill patients at a time, although optimally a separate nurse should be provided for each patient [4]. Such a situation, where the nurse/patient (N/P) ratio is higher than stated in international recommendations, poses a serious risk to the quality and safety of patient care and overburdens staff [5].

The direct impact of staff shortages is reflected in increased patient safety risks and impaired treatment outcomes [6]. Scientific studies have shown that excessive workload for nurses in ICUs has a negative impact on patients’ health [6,7]. Under high-workload conditions, the incidence of adverse events such as errors, infections and even mortality, increases [7]. A systematic review found [8] that higher nursing workload in ICUs is statistically associated with higher patient mortality risk and more adverse events. At the same time, overburdening also affects the staff, and prolonged activity in understaffing conditions contributes to professional burnout and staff turnover [8]. For example, studies in similar healthcare environments, including in Belgium, have shown that high nursing workload correlates with accelerated attrition of nurses and lower patient care outcomes [9]. Similar tendencies are also observed in Latvia. ICU managers point out that constant tensions and overburdening contribute to the departure of young nurses from the sector, with mostly older nurses staying at work [10]. This leads to a vicious circle where the quality and safety of care deteriorate due to insufficient staff, while the complexity of care and stress levels make it difficult to attract new specialists and retain existing ones [11].

In recent years, much attention in the international literature has been paid to quantifying and optimising ICU nursing workload for the sake of patient safety [12]. One of the most widely used tools for measuring nursing workload in intensive care is the Nursing Activities Score (NAS) [12]. The NAS was developed in 2003 by D. Miranda and co-authors as an improved alternative to previous evaluation systems such as TISS-28, and it has gained widespread international recognition, reaffirming reliability and practical applicability in intensive care environments in studies in different countries [13]. The NAS allows the amount of nursing care to be quantified, expressed as a percentage of the time of one shift. For example, 100 NAS points correspond to a situation where a shift of one full-time nurse is fully required to care for a patient over 24 h [13]. According to the latest meta-analysis summarising 70 observational studies with >56,000 patient data, ICU patient care globally requires about two-thirds of one nurse’s shift on average or ~66% of the workload per patient [12]. Moreover, NAS values tend to be even higher in certain periods, such as during patient admission and morning hours, and the average workload increased significantly during the COVID-19 pandemic compared to the pre-pandemic period [12]. This data challenges the conventional assumption that a fixed N/P ratio (for example, 1:2) is sufficient for an ICU. In fact, the average level of patient care intensity is closer to that of nearly 1:1 care, especially in case of severely ill patients and in crisis situations [14]. International experts therefore call for flexible staff planning models that take into account actual care needs, such as dynamic redeployment and reinforcements in situations where NAS values are above normal workload [15]. Traditional fixed staffing standards in many places no longer guarantee adequate care, and several countries are revising staffing principles based on objective workload measurements [16].

While the NAS has been widely applied internationally [14,15], most published studies are limited to single units, single hospitals, or short observation periods. Large-scale, nationally coordinated NAS datasets remain relatively scarce [14], particularly from smaller healthcare systems facing chronic nursing shortages. As a result, empirical evidence on how NAS performs across different ICU levels and institutional contexts under sustained workforce constraints is still limited. Studying nursing workload in such settings provides an opportunity to examine the robustness and interpretability of NAS under real-world pressure conditions, thereby contributing not only local but also internationally relevant insights into workload-based staffing models.

Latvia’s situation has so far been understudied compared to the international context. Although Western countries have developments in nursing workload monitoring and far-sighted staff planning, systematic measurement of nursing workload has not yet been implemented in Latvia [4]. The NAS was chosen as an appropriate solution for the intensive care needs of Latvia, as it covers approximately 81% of all nursing activities in intensive patient care compared to the older TISS-28, which covered only ~43% [5]. It is important that the NAS assessment is universal and independent of patient diagnosis, severity of disease or specific ICU profile [13]. This means that NAS results are comparable on a large scale and allow an objective assessment of how much patient care nurses actually perform during their working hours. The experience of foreign countries has shown that integrating such an objective workload measurement approach into staff management makes it possible to better balance the number of nurses with patients’ needs, prevent overburdening and improve the quality of care [9]. In other words, nursing workload data can serve as a basis for evidence-based decision-making, such as optimal measurement of the nurse/patient ratio in a particular unit, reallocation of work tasks or introduction of support mechanisms to ensure patient safety and better treatment outcomes [15,16]. The existing international literature often implicitly assumes that the highest nursing workload is concentrated in tertiary-level ICUs [9,10,11,12,13,14,15,16], reflecting greater patient acuity and technological intensity. However, empirical evidence comparing workload patterns across ICU levels remains inconsistent. In particular, less attention has been paid to intermediate-level ICUs, where staffing models, patient turnover, and care intensity may create unique workload profiles. Addressing this gap is important for understanding whether conventional assumptions about workload distribution across ICU levels hold true in different health system contexts.

In view of the above, it is clear that there is an acute need in Latvia for studies that evaluate the nursing workload in ICUs and allow for comparison with international data. So far, the country has lacked empirical data to quantify the size and structure of nursing workloads in intensive care units [4,5]. The intention is to bridge this knowledge gap with a study, which collects NAS measurements in several hospitals in Latvia in a uniform and systematic manner for the first time. Beyond addressing a national evidence gap, this study also enables comparison with international NAS-based research and tests commonly held assumptions regarding workload distribution across ICU levels and staffing models under conditions of sustained workforce shortage. Within the framework of the study, 28,079 observations were performed over 6 months in 14 ICUs across Latvia, filling the NAS, which provides the broadest insight so far into the nursing workload on a national scale. This data analysis will make it possible to assess actual trends in care intensity in Latvia, compare them with published data elsewhere in the world and identify critical discrepancies between available staff resources and patient needs. A scientifically sound understanding of nursing workload in ICUs in Latvian circumstances is important both in theory and in practice, as it facilitates a wider comparison of international and national contexts, strengthens the theoretical rationale for staff planning and serves as a reference point for further reforms in the healthcare system.

Finally, the study provides a reasoned basis for measures aimed at strengthening staff capacity in ICUs, improving the quality and safety of patient care and ensuring better treatment outcomes in the long term.

## 2. Description of Latvian ICUs

In Latvia’s healthcare system, ICUs form a structural and functional basis for providing acute care to patients with severe, life-threatening medical conditions. To ensure a differentiated approach to the treatment of patients and efficient use of resources, ICUs in medical treatment institutions in Latvia are divided into three levels [17]. Such classification is based on internationally recognised criteria and is defined in the guidelines of the Health Inspectorate and the National Health Service [18].

Level one ICUs are suitable for patients with moderate severity of life function impairment requiring short-term intensive monitoring and treatment, without significant support requirements for organ functions [19]. Level two ICUs provide treatment for patients with more serious conditions, including the need for artificial lung ventilation, invasive monitoring and long-term infusion therapy [19]. Level three ICUs, usually based in university hospitals or large regional hospitals, offer the highest intensity of care, including complex multiorgan support, dialysis therapy, long-term ventilatory support, and 24/7 access to specialist doctors and nursing staff [19]. Although ICU levels are formally defined according to clinical and technological criteria, staffing models and nursing task distribution may vary substantially between levels. In particular, intermediate (level two) ICUs often combine high patient turnover, complex monitoring, and limited access to specialised support compared with tertiary centres. These structural characteristics may result in high nursing workload that is not immediately apparent from patient acuity classification alone, underscoring the importance of empirically assessing workload across ICU levels rather than relying solely on formal level designation.

Nursing staff in each ICU play a central role in patient care regardless of the level. In Latvia, a person may work as an ICU nurse if he or she is a medical practitioner who has acquired at least first-level professional higher education (at college or bachelor level) in nursing studies, has been registered in the Register of Medical Practitioners and, preferably, has acquired specialisation in anaesthetic and intensive care [20]. Specialisation is achieved through graduation of professional improvement programmes harmonised with the requirements of the Cabinet of Ministers (CM) [20]. In addition, a nurse is obliged to renew his or her registration every five years, showing the number of hours of professional continuing education, thus ensuring that their knowledge and skills are in line with the development of the sector [21].

ICU nurses in Latvia are responsible for the entire cycle of patient care, from patient intake and monitoring of vital functions to performing complex procedures, administering medication, providing ventilatory care, communicating with patients and their loved ones, and maintaining care records [20]. Such specificity of care requires high professional competence and the ability to make clinical decisions quickly in complex and evolving situations. Although internationally the optimum N/P ratio in intensive care environments is considered to be 1:1–1.5, in Latvia’s reality this ratio is most often 1:2 or—even worse—1:3 or 1:5, especially at night or during holidays [19]. This disproportion increases the risk of professional error and reduces the quality of patient care.

In addition to registered nurses, several layers of support staff are also involved in the ICU care process. One of them is a medical assistant—a middle-level medical practitioner who is entitled to perform certain manipulations, as well as assist the physician in certain procedures. However, the medical assistant is not responsible for independent assessment or clinical decision-making for intensive care patients and only acts under the supervision of a registered nurse or physician [22].

Nursing assistants, who may not be medical practitioners, in particular students, but have acquired professional qualifications in the field of care, play an important role in practical care. Their duties include patient hygiene, positioning, mobilisation assistance and maintenance of the environment. While nursing assistants perform essential support tasks, they do not have the right to perform medical manipulations or document a patient’s treatment process. Their activities are always based on instructions and take place under the supervision of a registered nurse [23].

Some medical treatment institutions also employ physician assistants who have specific qualifications for performing medical support functions under the supervision of a physician. In the context of intensive care, their role is limited, and this profession is much less common in the units. When they are involved, their tasks are usually not limited to documenting or supporting certain procedures under the guidance of a physician, but they are trained, work and provide care as nurses [23].

The division of responsibility in ICUs is strictly regulated by laws and regulations, including the Medical Treatment Law [23], the Law on the Rights of Patients and the CM Regulations on classification of medical practitioners’ professions and care quality standards [20,22]. In accordance with this regulation, patient care is the responsibility of a treating physician and a registered nurse [23]. The performance of each care activity must be adequately documented and, where an element of care is delegated to another person such as a nursing assistant, the responsibility for supervising it remains with the nurse. This means that the legal responsibility for the care provided to the patient is directly linked to the boundaries of competence, professional standards and documented decisions of a medical practitioner.

The work organisation in Latvian ICUs is usually based on a shift system where 24/7 care is provided. During day and night shifts, the unit has a certain number of nurses, each responsible for a certain number of patients. Normally, there is also one senior or coordinating nurse in each shift who is responsible for staff allocation, work coordination, communication with physicians and continuity of care, but very often this function is performed by the unit’s head nurse. In level three ICUs, this structure is more detailed and multidisciplinary, while in lower-level units the activities tend to be more flexible, but with higher staff workload per patient in care [19].

Such organisational and legal context forms the basis for the quality of the care process and patient safety in the ICU environment of Latvia. At the same time, it marks significant challenges related to insufficient staffing, high workload and the need for structured, data-driven staff planning, especially in level three care centres. In such a regulated yet resource-constrained environment, objective workload measurement tools that are independent of diagnosis and unit profile are particularly relevant, as they allow nursing care demands to be quantified beyond formal staffing norms and institutional classifications. It is for this reason that studies [4,5], which systematically analyse the real care workload based on internationally recognised tools such as NAS, become relevant, allowing an assessment of the extent to which the provision of human resources in Latvia corresponds to the actual care needed by patients.

## 3. Materials and Methods

### 3.1. Study Design and Purpose

This study was developed as a quantitative, multicentre, observational study with repeated cross-sectional measurements aimed at assessing the workload of intensive care nurses in medical treatment institutions of different levels in Latvia using a structured assessment tool (NAS). The main objective of the study was to quantitatively analyse nursing care activities over a six-month period, identifying potential levels of overburdening, structural differences between units and possible discrepancies between available human resources and patients’ needs.

The study focused on examining the association between staffing adequacy and nursing workload, not only recording the actual intensity of care (measured in NAS points), but also assessing the alignment between available nursing staff and estimated care demands per shift. Thus, this study provides the first systematic set of data on the nursing workload in intensive care settings in Latvia at the national level, based on the use of an internationally recognised tool in everyday practice.

An important stage in the preparation of the study was the comprehensive training of the staff of interested hospitals in the use of the NAS to ensure a common understanding and consistency of data input. A three-month training cycle led was organised before the beginning of the study. Nurses, unit managers and responsible coordinators of all the ICUs involved attended this targeted training. The training content included the theoretical rationale of the NAS, interpretation of specific care activities, practical application, analysis of examples and simulation situations. As a result, it was ensured that the NAS was practically completed consistently and in line with methodological standards.

The study design was aimed at making the findings reflect the real circumstances of clinical practice without fostering interference of data collection with the care process. The nurses collected the information as they assessed each patient on the NAS based on the care activities carried out in the last 12 h. The data was collected in all the units involved in the study at the same time over a certain period of time, ensuring comparability between different regions, hospital levels and care practices.

### 3.2. Analysis Set and ICU Included

A total of 14 ICUs from various Latvian medical treatment institutions participated in the study, representing all 3 intensive care-level categories, as well as hospitals of different regional and institutional profiles. The selection was based on the principle of voluntary participation by inviting ICUs, which expressed interest in targeted workload assessments and were prepared to integrate NAS recording into the day-to-day care process. The set of units included provides representative coverage, both geographically and structurally, including both university hospitals, national-level hospitals, and also regional and local medical treatment institutions. Five level three ICUs, five level two ICUs and four level one ICUs were represented in the study. Three of the ICUs had mixed-type beds, and had both level 2 and level 3 beds.

Such an analysis set made it possible not only to obtain quantitative amounts of data, but also to compare the intensity of care, staff workload and organisational differences in care environments of different levels. The included units covered both Riga territory and regional hospitals in Kurzeme, Vidzeme, Zemgale and Latgale, thus ensuring comparability of different care models and available resources.

All the units included had a pre-structured composition of care staff, where staff completed NAS as part of their daily shift documentation. In all the hospitals, data entry was performed digitally, directly in electronic form, using customised NAS forms.

Overall, the selected sample model ensures good internal and external comparability and enables analysis of both the overall workload trend and the differences between units, regions and intensive care levels. In subsequent analyses, this diversity was used to identify structural and organisational factors that may affect the extent and intensity of care workload.

As ICU participation was voluntary, the sample may not represent all ICUs nationwide; however, the inclusion of units from all three ICU levels and all major regions of Latvia provides broad structural and geographical coverage.

### 3.3. Data Collection

Data was collected over a six-month period from 01.05.2025 to 01.11 2025 in all 14 ICUs involved in the study, covering treatment institutions of different levels throughout Latvia. NAS observations were recorded at the shift level and may include repeated measurements within the same unit and across patients; therefore, the data structure reflects clustered observations at the unit level rather than independent patient-level measurements. The nurses registered in each unit monitored patients during each 12 h shift and completed NAS forms based on the care activities performed during that shift. This approach ensured the reliability of the data and at the same time had minimal impact on the flow of daily clinical care.

Data were only collected electronically, and all calculations were made automatically. Recording was performed for all patients who received active intensive care during the given shift, and 28,079 NAS observations were obtained during six months, making this the largest workload dataset to date in intensive care in Latvia.

In total, staff from 14 ICUs were involved in the data collection, with 369 registered nurses, 32 physician assistants, 48 medical assistants directly completing NAS and observing and 115 assistant nurses responsible for patient hygiene, care environment and technical support.

This staff distribution reflects the multi-layered care model of intensive care in Latvia, where much of the practical care is organised under the guidance of a nurse, involving support staff as part of the delegation of tasks.

A total of 167 intensive care beds, 29 of which were in level one ICUs, 59 were in level two ICUs and 79 were in level three ICUs, were registered in the study.

Shift work was used in all the units—most often two 12 h shifts per day (for example, 08:00–20:00 and 20:00–08:00)—however, 24 h shift was still used in many places. During data collection, staff distribution by workdays, weekends and public holidays was also documented, allowing for later analysis of workload fluctuations between different periods.

### 3.4. Tool Used in the Study

The NAS was used in this study to objectively assess the nursing workload in ICUs. It is one of the most internationally recognised tools for measuring care intensity [12,13]. The NAS was developed as an improved alternative to TISS-28, with a particular focus on the specificity of nursing functions and quantifying workload in the context of intensive care. The NAS includes 23 items that cover the most frequent nursing activities and allow the intensity of care to be expressed as a percentage. The NAS value is 100% equivalent to the full-time work of one nurse with one patient during 24 h.

Each item on the NAS corresponds to a specific care activity with an assigned percentage (for example, respiratory support, mobilisation, discharge organisation), allowing real-time documentation of care intensity and forecasting of the required staff resource. This feature makes the NAS particularly suitable for both operational workload management and long-term systemic analysis.

Prior to the commencement of this multicentre study in Latvia, a pilot study was carried out in three ICUs [4] aimed at assessing the suitability of the NAS tool for the local context. As part of the adaptation, terminology was tested, staff training was performed and first experience of usability of the tool was acquired. While the scale of the study was limited, it allowed the practical challenges to be identified and application algorithms to be improved before the wider rollout.

### 3.5. Data Processing and Statistical Analysis Methods

Upon completion of the data collection, all acquired NAS protocols were centrally collected and prepared for further analysis using IBM SPSS Statistics for Windows, version 20.0 (IBM Corp., Armonk, NY, USA). The data processing process involved a sequence of several steps, ranging from quality control and data purification to analysis of descriptive and comparative statistics.

A total of 28,154 NAS protocols were obtained, of which 28,079 complete records were included following manual inspection and quality control. Excluded cases mainly included entries with missing data on the type of shift, NAS total or other relevant variables. Each protocol included information about the hospital code, unit level (Level 1, 2 or 3 ICU), type of shift (day or night shift), total NAS points value, as well as the number of staff and calculated patient/nurse ratio. A staff deficit indicator was also included if the data were available.

As part of the descriptive statistics, mean NAS points, standard deviation, median, quartile (Q1, Q3), interquartile range (IQR) and minimum and maximum values were calculated for each unit and type of shift. These indicators made it possible to characterise the intensity of care in different clinical contexts and to identify variability between treatment institutions.

Several inferential tests were performed to assess statistically significant differences. The independent samples *t*-test was used to compare NAS points between day and night shifts, while one factor analysis of variance (ANOVA) was used to compare mean NAS values between level one, level two and level three ICUs. In cases where ANOVA showed statistically significant differences, a Tukey’s HSD post hoc test was applied to identify specific sources of differences between groups. In addition, a Pearson correlation analysis was performed to assess the relationship between NAS point values and the patient/nurse ratio or other workload indicators. Given the large sample size, statistical significance was interpreted in conjunction with effect sizes and clinical relevance, and small *p*-values with negligible effect sizes were interpreted cautiously.

The results were supplemented with visuals, including boxplot charts, to illustrate NAS distribution across different units and shift types, as well as line graphs to analyse care workload dynamics over time. This combined approach made it possible not only to identify structural differences between medical treatment institutions, but also to assess the variability of care intensity according to time of day or month.

Staff shortages were calculated by comparing the actual number of nurses available on a given shift with the theoretical need, which was obtained on the basis of the sum total of NAS points. If the level of actual supply was lower than required, it was classified as a staff shortage and expressed as a percentage. These calculations were made for each medical treatment institution, each month and each type of shift, providing a detailed view of the adequacy of human resources to care needs.

Time-series analysis was conducted to explore temporal patterns in aggregated NAS values; details of the modelling approach are provided in the Results section.

### 3.6. Ethical Considerations

This study was carried out in accordance with the principles of the Helsinki Declaration [24] and the current Latvian regulatory framework for the protection of human data and research ethics. A permit was obtained from the Ethics Committee of Rīga Stradiņš University (protocol No. 2-PEC-4/416/2023) prior to the beginning of the study, which confirmed that the methodology of the study complied with ethical requirements and subjects’ rights were fully respected.

The study did not collect personally identifiable information, thus ensuring anonymity and confidentiality of respondents at all stages of the study. NAS protocols were completed using unique hospital and shift identifiers that prevent specific individuals from being identified. All the findings were stored in a protected environment with access only by those involved in the study.

Participation in the completion of the data was organised on the basis of informed consent, and all the nurses involved in the study were informed in advance of the purpose of the study, the data usage procedure and the principles of voluntary participation. The data collection did not affect staff work or clinical care, and there was no intervention in the patient treatment process.

During the study, the procedures for division of responsibility and organisational coordination were also followed. The management of each hospital gave formal permission to carry out the study and assigned responsible contacts to collect and transmit NAS data. After analysis of the data, the results will only be used for scientific and policy planning purposes, without the possibility of detecting individual- or institution-specific sensitive indicators.

## 4. Results

### 4.1. Descriptive Statistics

The mean NAS across the analysis set was 65.45 points (SD = 25.76; median = 60.8; range 10.20–176.8), indicating high care intensity, whereby care for one patient during a 12 h shift requires approximately two-thirds of a nurse’s workload on average. The observed range reflects substantial variability in workload across shifts and units, from low-intensity care to situations in which a single patient required more than a full nurse workload per shift. No missing NAS or staffing indicators were observed, as all protocols were completed digitally using mandatory fields.

When analysed by level of care, mean NAS values were 52.46 points (SD = 23.98) in level 1 units, 79.60 points (SD = 27.97) in level 2 units, and 64.10 points (SD = 23.09) in level 3 units, indicating the highest average workload in level 2 ICUs.

Marked interunit variability was observed, with the lowest mean NAS recorded in unit 7 (M = 38.77) and the highest in unit 1 (M = 86.41). In several units, NAS values exceeded 100 points, indicating that care for a single patient required more than one full nurse workload per shift.

Descriptive statistics by unit are summarised in Table 1, with the full version provided in the Supplementary Materials. Summary statistics by level of care are presented in Table 2 (full version in the Supplementary Materials). Of all recorded protocols, 16.5% were from level 1 units, 21.1% from level 2 units, and 62.4% from level 3 units.

Descriptive patterns by day of the week are summarised in Table 3 (full version in the Supplementary Materials). Mean NAS values were relatively stable across weekdays, with slightly higher values observed on Sundays and public holidays.

**Figure 1:**
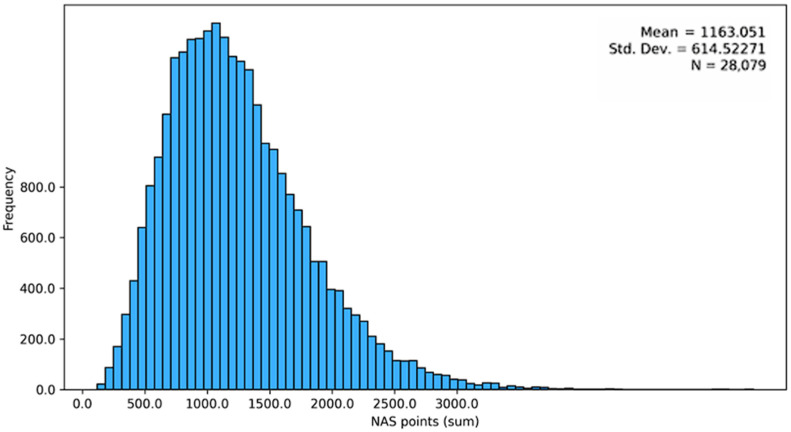
Histogram of NAS totals.

Across the full dataset, the total mean NAS per shift was 1163.05 points (SD = 614.52; median = 1148.30), with a wide distribution reflecting substantial variation in aggregated care demand. Given the large sample size, deviations from normality were expected; therefore, parametric test results were interpreted alongside effect sizes and robustness checks (Figure 1).

#### Number of Patients

The mean number of patients was 10.28 (SD = 5.50; median = 10.00) during day shifts and 9.43 (SD = 5.70; median = 8.00) during night shifts (Supplementary Materials, Figure S1).

**Figure 2:**
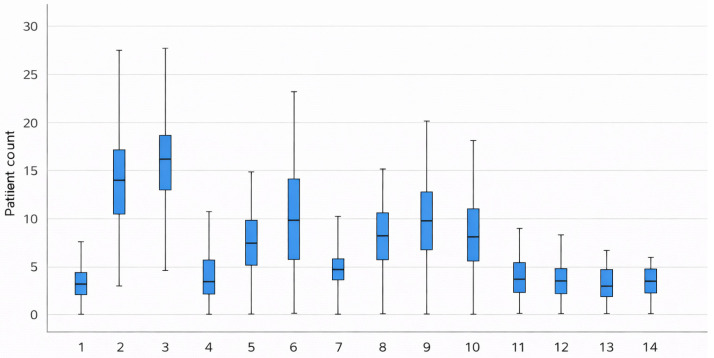
Boxplot for the number of patients in 14 ICUs.

Substantial variation in patient numbers was observed across ICUs. Unit 3 had the highest average number of patients per shift (M = 15.86; SD = 4.75), whereas units 1, 4, 11 and 12 consistently reported fewer than five patients per shift. The largest variability was observed in unit 9 (range = 18), while the smallest was observed in unit 13 (range = 7). This heterogeneity is illustrated in Figure 2.

### 4.2. Comparative and Inferential Analysis

#### 4.2.1. NAS Differences Between Shifts, Units, Levels and Days of the Week

Mean NAS values did not differ meaningfully between day and night shifts (*p* = 0.216; Cohen’s d = 0.015), indicating comparable workload intensity across shifts. The distribution of NAS values by shift type is illustrated in the Supplementary Materials (Figure S2).

**Figure 3:**
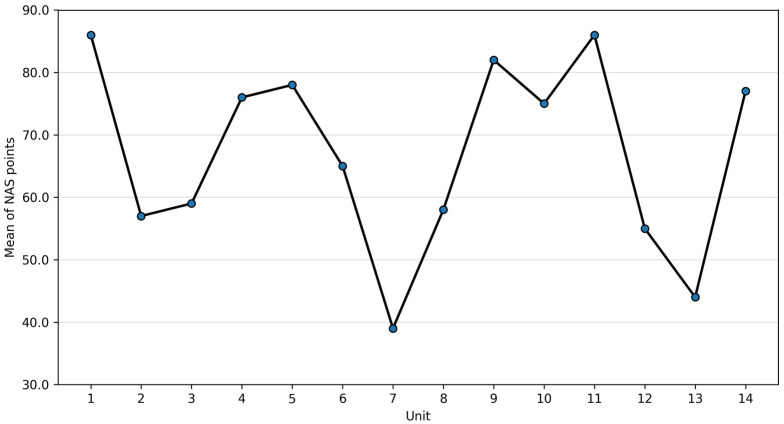
Mean NAS by unit (95% CI).

Significant differences in NAS were observed between intensive care units (one-way ANOVA: F = 650.70, *p* < 0.001), with a large effect size (η^2^ = 0.232), indicating that approximately 23% of the variance in workload was attributable to unit-level differences. Mean NAS values varied widely across units, with the highest and lowest values shown in Figure 3.

NAS also differed across levels of care. Mean NAS values were lowest in level 1 units (M = 52.46; SD = 23.98), highest in level 2 units (M = 79.60; SD = 27.97), and intermediate in level 3 units (M = 64.10; SD = 23.09). Differences between levels were statistically significant (F = 248.13, *p* < 0.001), although the effect size was small (η^2^ = 0.017). Post hoc comparisons indicated that level 2 units exhibited higher workload intensity than both level 1 and level 3 units.

**Figure 4:**
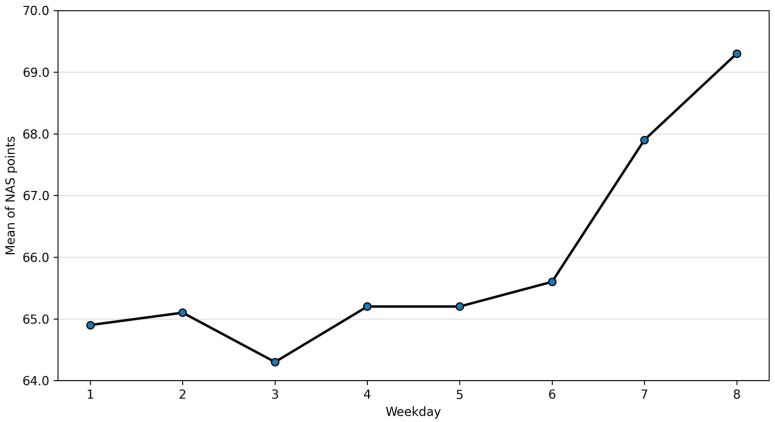
Mean NAS across the week (95% CI).

Descriptive patterns suggested modest variation in NAS values across the week, with slightly higher values observed on Sundays and public holidays (Figure 4).

Results were robust to heteroscedasticity, as Welch ANOVA yielded conclusions consistent with the main ANOVA findings. Overall, comparative analyses indicate that workload differences were driven primarily by unit-level and care-level characteristics, while differences between shifts were negligible.

#### 4.2.2. Mixed-Effects Model and Interunit Variation

To further quantify structural sources of workload variation, a linear mixed-effects model was fitted with ICU as a random intercept and level of care, shift, and weekday as fixed effects. The model demonstrated a significant unit-level effect (Wald Z = 10.41, *p* < 0.001), with an intraclass correlation coefficient of 0.214, indicating that approximately 21.4% of total NAS variance was attributable to differences between units.

Among fixed effects, level of care and weekday were statistically significant, whereas shift type showed no meaningful association with NAS values. Model fit was substantially improved compared with a fixed-effects model (ΔAIC = −1540), confirming that a substantial proportion of workload variability reflects structural and organisational differences between ICUs rather than shift-specific factors.

#### 4.2.3. Care Time per Patient per Day

When expressed as equivalent nursing care time, where 100 NAS points correspond to 24 h of nursing work, the mean care time per patient was 15.71 h per day (SD = 6.18; range 2.45–43.58). This corresponds to approximately 65% of a full-time nurse per patient during a 12 h shift.

Care time did not differ meaningfully between day and night shifts, reflecting the continuous nature of intensive care. However, substantial variation was observed between units, with average care time ranging from 9.30 to 20.74 h per patient per day. Unit affiliation explained approximately one-fifth of the variance in care time (η^2^ ≈ 0.18).

#### 4.2.4. Care Structure (NAS Domains)

Analysis of NAS domains showed that Basic activities accounted for the majority of nursing workload (71.7% of total NAS), followed by Renal (12.0%) and Ventilatory care (6.3%). Cardiovascular, Metabolic, Specific, and Neurological activities each contributed smaller proportions to overall workload.

Among domain-level associations, the strongest correlation was observed between Ventilatory and Metabolic activities (r = 0.34), while other inter-domain correlations were weak, indicating that workload components were largely independent (Supplementary Materials, Figure S3).

Domain-level regression analysis indicated that NAS domains collectively explained a modest proportion of variance in total workload (R^2^ = 0.079), suggesting that overall NASs are driven by the cumulative contribution of multiple care activities rather than a single dominant domain.

#### 4.2.5. Nursing Shortage and Relation to NAS

**Figure 5:**
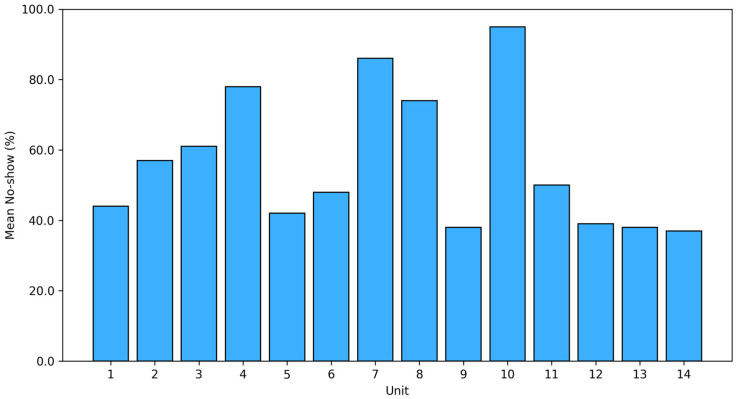
Column chart: nursing workload by unit.

The average nursing shortage across all observations was 42.58% (SD = 44.98), indicating substantial staffing deficits. Shortage levels differed markedly between units (F = 377.89, *p* < 0.001; η^2^ = 0.149), ranging from 4.51% to 63.34% (Figure 5).

Nursing shortages were slightly higher during night shifts than day shifts; however, the effect size was negligible, indicating limited practical significance.

Shortage levels also differed across levels of care, with higher shortages observed in level 1 and level 2 units compared with level 3 units.

Linear regression analysis demonstrated a significant association between NAS workload and nursing shortage (R^2^ = 0.115). Higher NAS values were associated with greater staffing deficits, underscoring a structural mismatch between care demand and available nursing resources. The regression equation is

Nursing shortage (%) = 13.76 + 0.025 × NAS sum

This indicates that each additional NAS point was associated with a mean increase of 0.025% in nursing shortage.

In hierarchical regression, structural factors (change, care level) alone explained only 0.4% variation in shortage (R^2^ = 0.004), while summing NAS and number of patients increased R^2^ to 0.166 (ΔR^2^ = 0.162, *p* < 0.001). NAS was the strongest positive predictor (β = 0.522, *p* < 0.001), care level was secondary (β = 0.224, *p* < 0.001), but the number of patients showed small negative effect (β = −0.107, *p* < 0.001), while shift had no significant effect.

The real N/P ratio and the NAS-based required number of nurses per unit were calculated to further assess staff compliance. The results show a significant discrepancy between the actual amount of care resources and the amount of care resources needed. Table 4 summarises key indicators—actual number of nurses, number of patients, real N/P ratio, NAS-based estimated number of nurses required, staff shortages and Workload Index.

**Figure 6:**
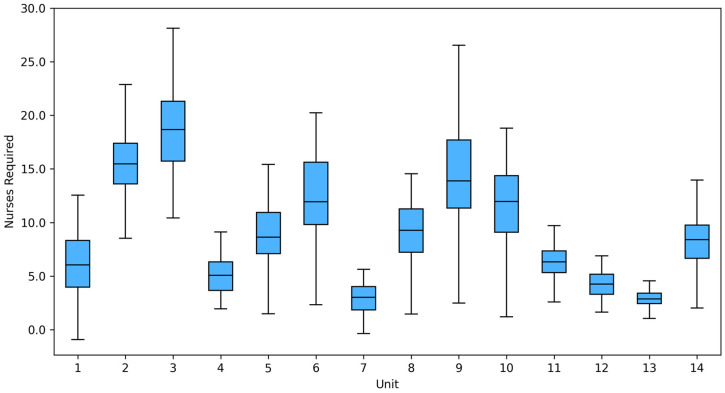
NAS-based distribution of nurses required (NursesRequired) in 14 ICUs.

Boxplots (Figure 6) were created to visualise NAS-based staff demand in various intensive care units. The results show significant differences between units: units 2, 3, 6, 9 and 10 show the highest average number of nurses required (≥15 per shift), while units 12, 13 and 14 show the lowest demand and lowest dispersion. 

#### 4.2.6. Relationship Between the Number of Patients, NAS, and Workload Index

The total number of patients was strongly associated with aggregated NAS workload (r = 0.84), whereas NAS per patient remained relatively stable across varying patient counts. Workload Index increased with patient volume and was significantly predicted by the number of patients (R^2^ = 0.31), although a plateau effect was observed at higher patient loads, suggesting capacity limits in staffing adjustment.

No significant correlation was observed between NAS workload and the actual number of nurses per shift, indicating that staffing levels were not systematically adjusted to fluctuations in care intensity.

#### 4.2.7. Z-Score Normalisation and Workload Heatmap

Standardised NAS values highlighted pronounced and consistent interunit differences in workload. Units 1 and 11 showed persistently higher workloads than the national average, whereas units 7 and 13 consistently exhibited lower workload levels. Weekly cyclical patterns were evident in several units, with higher workloads at the beginning of the week and relative reductions toward weekends. Level 2 units displayed consistently elevated workload across the weekly cycle:

(1) $$Z_{i}=\frac{NAS_{i}\;-\;65.45}{25.76}$$

**Figure 7:**
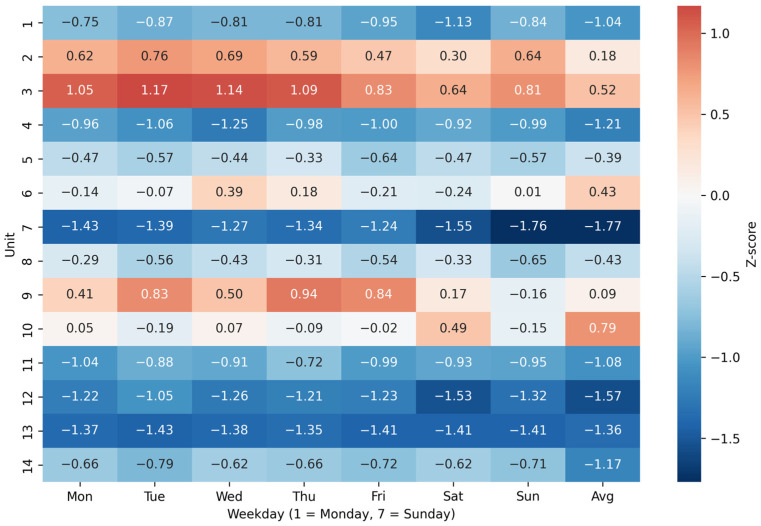
Heatmap for unit workloads.

Positive Z-score values (>0) indicate a higher workload than the national average; negative (<0)—a lower workload. The heatmap (Figure 7) shows the mean Z-score values in each of the 14 units over 7 days of the week.

### 4.3. Time Trends and Forecasts

#### 4.3.1. NAS Time Trend

To explore temporal dynamics in workload across the study period (May–October), we examined NAS values over time. A linear trend indicated only minimal change, explaining a negligible proportion of variance (R^2^ = 0.005), suggesting that overall workload levels remained broadly stable across months without a clinically meaningful long-term trend.

#### 4.3.2. Monthly Dynamics of Workload and Staff Sufficiency

Monthly summaries showed modest variation in aggregated workload and staffing sufficiency across the six-month period. Mean NAS workload was highest in May, decreased slightly during the summer months (June–August), and stabilised in autumn. Nursing shortages followed a similar pattern, with relatively higher deficits early in the study period and gradual improvement towards October. Differences between day and night shifts remained small across months.

#### 4.3.3. NAS and Workload Index Seasonality

**Figure 8:**
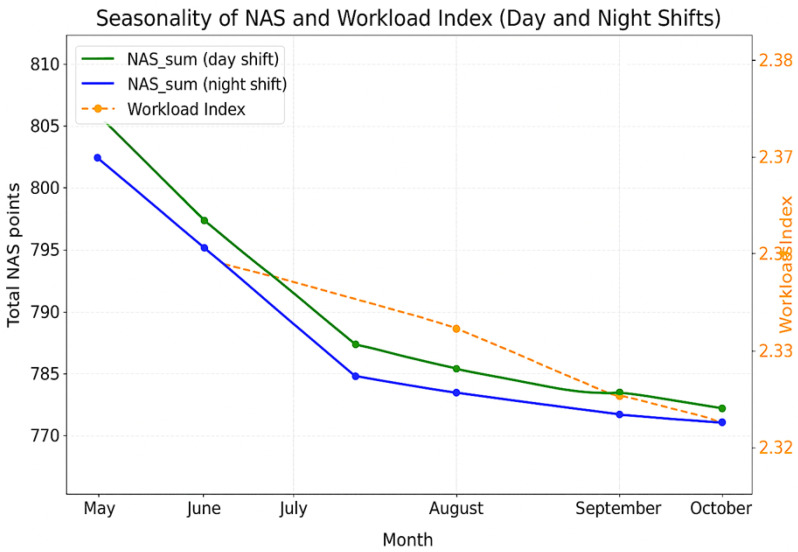
NAS and Workload Index seasonality.

Figure 8 illustrates monthly seasonality patterns in total NAS workload and Workload Index. NAS totals followed similar trajectories across day and night shifts, while the Workload Index remained relatively stable throughout the period, with only minor month-to-month fluctuations.

#### 4.3.4. Time-Series Analysis (ARIMA)

An ARIMA(1,1,16) model with NAS sum total as a dependent variable was used to predict NAS workload dynamics. The model showed good adjustment (R^2^ = 0.797), indicating that approximately 80% of NAS variation is explained by values of the previous period. Forecast errors were clinically acceptable (RMSE = 276.8; MAE = 126.3; MAPE = 25.4%). The autoregressive factor AR(1) = 0.875 (*p* < 0.001) indicated a strong dependence on the previous period, while MA components (MA(1), MA(2), MA(16)) were statistically significant, reflecting short-term fluctuations. The autocorrelation of balances varied within ± 0.02 and the chart showed a good correspondence between observed and predicted values. The projections suggest that the NAS workload will remain high in the near future, with a concentration of predicted values around 1100 ± 100 points.

The ACF chart showed a marked autocorrelation in the first lags (lag 1 = 0.56; lag 2 = 0.27), but then the correlations approached zero. The PACF’s first lag dominated the rest, evidence of AR(1) structure. The Box–Ljung test *p* < 0.001 confirmed that the time series is not white noise, and modelling is justified.

### 4.4. Additional Workload Indicators: Workload Index

**Figure 9:**
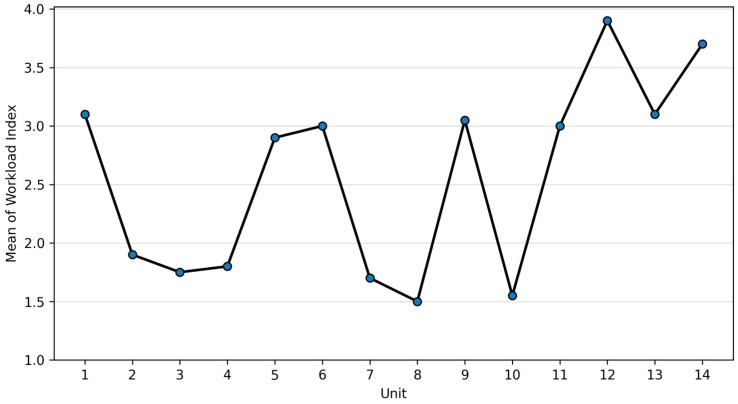
Workload Index distribution by unit.

Workload Index values differed between ICUs, with the highest indices observed in units 1, 12, 13 and 14, suggesting substantial overburdening, while comparatively lower indices were observed in units 7, 8 and 10 (Figure 9).

Workload Index was positively associated with aggregated NAS workload, although the relationship was weak overall (r = 0.221). Consistent with earlier findings, Workload Index also differed across levels of care, with higher values observed in level 1 and level 2 units compared with level 3 units (η^2^ = 0.148), suggesting greater staffing strain outside tertiary centres despite the clinical severity typically concentrated in level 3 ICUs.

Across shifts, Workload Index values were slightly higher at night; however, the practical difference was minimal. Month-to-month variation in Workload Index was statistically detectable but small and of limited clinical relevance.

**Figure 10:**
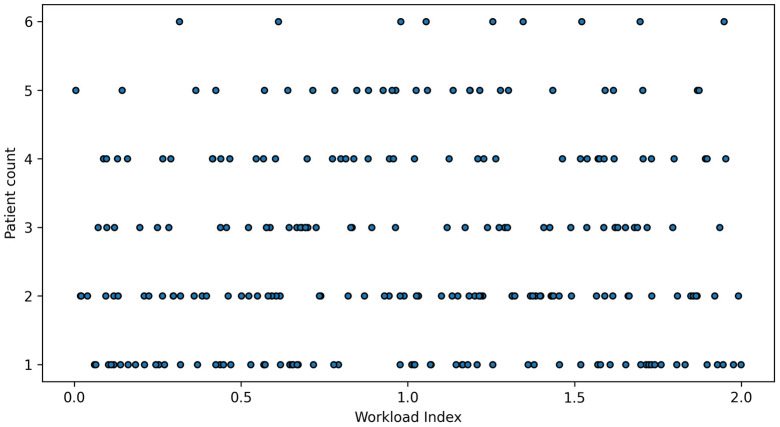
Relationship between number of patients and Workload Index.

Finally, Figure 10 illustrates the relationship between patient volume and Workload Index. While workload generally increased with patient numbers, substantial dispersion at similar patient counts suggests that workload intensity is influenced not only by patient volume but also by care complexity and organisational factors.

## 5. Discussion

This six-month multicentre study showed that the average time of care per intensive care patient in Latvia is 15.71 h per day, corresponding to approximately 65% of a full-time nurse’s workload. In practical terms, this indicates that under routine conditions one nurse can realistically provide full care to approximately 1–1.5 patients during a 12 h shift. These values are consistent with internationally reported NAS findings, where average daily care time typically ranges between 14 and 17 h [9,13], confirming that ICU nursing practice in Latvia is characterised by sustained high care intensity and continuous clinical engagement.

However, beyond confirming high overall workload, the present findings reveal substantial heterogeneity across units, care levels, and hospitals, with mean care time varying almost twofold between 9.30 and 20.74 h per patient per day. The observed effect size (η^2^ ≈ 0.18) indicates that approximately one-fifth of the variance in workload is attributable to unit-level factors, pointing to pronounced structural differences in staffing models, organisational arrangements, and patient flow rather than random fluctuation. Comparable interunit variability has been described in multicentre NAS studies from Belgium and other European contexts [9], suggesting that Latvia’s findings reflect a broader systemic pattern rather than an isolated anomaly. Nevertheless, the Latvian data highlight a particularly marked misalignment between care demand and staffing availability, indicating that human resource allocation is not proportionally adjusted to actual nursing workload across ICUs.

In several units, nurses were required to deliver care approaching a full-time workload for a single patient, while in lower-intensity units one nurse could care for multiple patients within the same shift. Such disproportions imply that nursing workload is distributed unevenly across institutions, exposing nurses in certain units to sustained overburdening that may substantially exceed normative expectations. Previous research has consistently linked excessive nursing workload to increased risk of burnout, missed care, adverse events, and compromised patient outcomes [9,25]. In the Latvian context—where the nurse-to-population ratio remains among the lowest in the European Union and shortages of specialised ICU nurses are chronic [3]—these findings underscore a heightened vulnerability of both staff well-being and care quality.

To appropriately interpret these findings, it is essential to clarify the conceptual role attributed to the NAS in the present study. NAS is not treated here as a proxy for nursing care complexity as defined by nursing diagnoses, clinical reasoning, or care planning processes. Rather, NAS is conceptualised as an indicator of realised care demand and organisational workload intensity, reflecting the cumulative volume and time consumption of nursing activities required to maintain patient stability within a given system context [26,27,28,29].

Accordingly, high NAS values should not be interpreted as direct indicators of patient risk or clinical severity [7]. Instead, they signal conditions of system strain, in which nursing resources are intensively mobilised to sustain essential care processes. In healthcare systems characterised by persistent staffing shortages, elevated NAS values may therefore reflect compensatory nursing effort rather than inherently higher patient acuity [11].

Previous empirical studies have demonstrated that nursing care complexity—operationalised through nursing diagnoses and nursing actions—predicts adverse patient trajectories independently of medical severity indicators. These findings capture qualitative dimensions of nursing work, including cognitive load, clinical judgement, and relational care, that are not fully encompassed by time-based workload instruments such as NAS. The present study does not seek to replace or contradict this body of evidence.

Instead, NAS-based workload and nursing care complexity are treated as analytically distinct but complementary constructs [9]. Nursing care complexity reflects the qualitative and decision-intensive aspects of clinical practice, whereas NAS captures how these demands translate into measurable workload within constrained organisational environments. High NAS values therefore represent a structural risk context in which nursing care complexity is more likely to exert downstream effects on patient outcomes and staff sustainability, even if NAS itself is not a direct predictor of such outcomes [25,26].

Applied to the Latvian ICU context, this conceptual framing suggests that persistently high NAS value, particularly in level 2 ICU, reflect organisational vulnerability rather than exceptional patient acuity. Intermediate-level units often combine high patient turnover, diverse clinical profiles, and limited staffing flexibility, creating conditions in which workload intensity escalates independently of formal ICU classification. This finding extends existing NAS literature by demonstrating that system strain may be most pronounced outside tertiary ICUs, challenging assumptions that workload is highest where technological intensity is greatest.

Analysis of the care activity structure further demonstrated that Basic activities accounted for the majority of nursing workload (71.7% of total NAS), with Ventilatory and Renal care contributing smaller but clinically significant proportions. This pattern is consistent with international NAS studies showing that general, continuous nursing care—rather than isolated technical procedures—constitutes the primary driver of workload in intensive care settings [13,26]. While often perceived as routine, these basic activities form the organisational substrate through which nursing care complexity is enacted in practice, particularly under conditions of high workload and limited staffing. Hygiene, positioning, communication, symptom management, and continuous observation require sustained attention and coordination, contributing substantially to cumulative workload.

The multidimensional structure of NAS was supported by weak correlations between most care domains, indicating that workload is generated through the aggregation of diverse care activities rather than domination by a single task category. Certain activity combinations—such as ventilatory and metabolic care or cardiovascular and renal care—formed physiological workload clusters typical of patients with multiorgan dysfunction. These patterns illustrate that Latvian ICU nurses are required to balance technical interventions with prolonged basic care within the same shift, reinforcing the complexity of real-world care delivery even when complexity is not directly measured by NAS [30].

Seasonal analysis revealed moderate but consistent fluctuations in workload and staffing shortages, with higher demands observed in spring, partial relief during summer months, and renewed increases in autumn. These trends align with international evidence linking ICU workload variation to seasonal morbidity patterns and elective surgical activity [9,27]. In Latvia, such fluctuations likely interact with staff leave periods, further exacerbating workforce constraints during predictable high-demand intervals.

Importantly, NAS values did not differ meaningfully between day and night shifts, indicating that intensive care workload remains continuously high regardless of time of day. Although small statistical differences were observed in nursing shortage rates and Workload Index values, their practical impact was negligible. These findings reinforce the principle of care continuity in intensive care and challenge assumptions that night shifts are intrinsically associated with lower workload intensity.

Time-series modelling provided further insight into workload dynamics. The ARIMA(1,1,16) model revealed a strong autoregressive component (AR(1) = 0.875), indicating that NAS values are highly dependent on previous periods. This structural inertia suggests that ICU nursing workload is not randomly volatile but persistently high and predictable, supporting the need for proactive, data-driven workforce planning rather than reliance on historical staffing patterns.

The relationship between workload and staffing adequacy was particularly pronounced. Regression analyses demonstrated that nursing shortages were primarily driven by NAS workload, whereas structural variables such as shift type or ICU level explained only minimal variance. This indicates that staffing deficits arise when objectively measured care demand exceeds available nursing capacity, rather than as a consequence of scheduling conventions or unit classification. The association was strongest in level 2 ICUs, where mixed patient profiles and limited staffing buffers may amplify system strain.

From a managerial and policy perspective, these findings challenge the adequacy of traditional staffing heuristics based on fixed nurse-to-patient ratios or unit typology. Objective workload indicators such as NAS provide a more accurate representation of care demand and should therefore inform staffing decisions. This aligns with international recommendations emphasising the limitations of fixed ratios under conditions of complex care delivery and workforce scarcity [9].

Within the broader Latvian healthcare context—characterised by a low nurse-to-population ratio, ageing workforce, and difficulties in attracting and retaining ICU nurses—persistently high workload levels pose a substantial risk to both patient safety and staff sustainability. Previous Latvian and international studies have already linked such conditions to burnout, moral distress, and potential workforce attrition among intensive care nurses.

Overall, this study demonstrates that ICU nursing workload in Latvia is not only high but structurally uneven, predictable, and closely linked to organisational capacity constraints. Nursing shortages are primarily driven by objectively measurable workload rather than by administrative or temporal factors. These findings provide a robust conceptual and empirical foundation for integrating NAS-based workload monitoring into system-level workforce planning, while recognising its complementary role alongside nursing-sensitive measures of care complexity.

## 6. Proposals

The results of this study demonstrate that the workload of intensive care nurses in Latvia is consistently high and structurally heterogeneous, while available staffing levels frequently do not correspond to actual care intensity. The almost twofold variation in workload between units indicates a systemic imbalance in human resource allocation and highlights the need for data-driven workforce planning tools.

First, the findings support a shift from fixed nurse-to-patient ratios toward dynamic staffing models based on objectively measured care intensity. The average care time of 15.71 h per patient per day indicates that fixed ratios are insufficient under high-intensity conditions and may compromise care safety during peak workload periods. Incorporating NAS-based workload indicators into staffing decisions would allow a more accurate alignment of nursing resources with real-time care demands.

Second, the introduction of a unified NAS-based workload monitoring system across ICUs is recommended. Such a system would support objective identification of units experiencing critical staff shortages, facilitate interunit resource balancing, and provide a transparent basis for managerial decision-making. Centralised NAS monitoring could also enable early detection of workload peaks and support proactive staffing adjustments.

Third, the observed seasonal workload fluctuations indicate that workforce planning should account for predictable changes in patient flow and staff availability. Integrating seasonal workload projections into annual staffing plans may reduce periods of simultaneous high workload and reduced staff availability, particularly during spring and early summer.

Finally, persistently high workload levels observed in several units underline the importance of parallel investments in staff well-being and retention. Sustained exposure to near-maximum workload increases the risk of burnout, moral distress and workforce attrition. Therefore, workload monitoring should be complemented by organisational measures aimed at supporting staff resilience and long-term workforce sustainability.

Overall, these proposals emphasise that effective intensive care workforce planning requires objective workload measurement, dynamic staffing models and system-level coordination. Adoption of NAS-based approaches may contribute to safer patient care, improved working conditions for nurses and greater sustainability of intensive care services in Latvia.

## 7. Limitations

The interpretation of this study should be considered in light of a number of methodological and practical limitations which may affect the generalisation of results and their potential application in a broader context. Although the scope of the study data was considerable and covered several ICUs throughout Latvia, it nevertheless reflects the situation over a specific period of time and under certain organisational conditions. NAS measurements were taken over a six-month period, and while this period allows seasonal fluctuations to be identified, it does not cover a full year and does not allow long-term cyclicality or perennial trends to be accurately determined. Data collection also took place in ICUs, which currently use different workflows and organisational models, which can lead to some heterogeneity in measurement practices.

Another significant limitation is that the NAS data were collected in real-life care settings, where nurses worked under high-workload conditions and could not always ensure a fully consistent completion of the score. Although all measurements comply with the NAS methodology, there is a likelihood that subjective assessment and lack of time may have affected the recording of individual activities. This limitation is inherent in all observational studies, which are based on routine clinical data, and does not imply a significant systematic error, but it should be taken into account in interpreting the results.

It should also be emphasised that the study does not cover data from the disease severity index (for example, SOFA or APACHE II), which would allow a more precise assessment of the extent to which the differences in workload between units are due to patient profiles rather than organisational factors alone. While NAS partly reflects patient severity, the disease severity indices would have allowed for a more detailed analysis of how specific clinical parameters affect the intensity of care needed. In addition, the study did not analyse patient outcome indicators that could provide an in-depth insight into the relationship between workload and quality of care.

It should also be mentioned that the study hospitals differ in size, profile, region and patient structure, which, while enriching the dataset, makes a completely uniform comparison between institutions difficult. Some units have consistently higher rates of care and more complex patients, while other units care for a wider group of less acute patients. These structural differences were not fully controlled because the primary objective of the study was to describe the reality of workload rather than isolate the effects of individual factors.

Time-series modelling should take into account that NAS and nursing shortage data were analysed without additional exogenous variables that could potentially affect the accuracy of the forecasts. The ARIMA model revealed structural time dependency, but the predictive capacity of the shortage was limited because external factors such as patient severity fluctuations, elective surgery activity, leave schedules, availability of backup staff or temporary eHealth and infrastructure malfunctions were not included in the model. This limitation is significant as it shows that shortage dynamics are more complex than a linear dependency on NAS workload and require a multi-factor approach to ensure accurate forecasting.

Another limitation is that soft indicators such as the well-being, burnout, moral distress of nurses or quality of work environment were not included in the study, although previous studies in Latvia and internationally have shown that they are closely related to the objectively measurable workload. The absence of such information limits the possibility of fully assessing the impact of different workload patterns on the psychological and emotional health of staff, which is an essential aspect of sustainability of intensive care.

It should also be noted that the study does not allow establishing a clear “cause and effect”. While the analysis clearly shows that higher NAS levels are closely linked to nursing shortages, the observational design does not allow the conclusion to be drawn as to whether shortages occur directly because of the increase in workload, or whether other factors, such as sudden employee absences, institutional policies or local organisational limitations, exacerbate this.

Finally, although the analysis set of the study is one of the largest in the history of NAS studies conducted in Latvia, it is still not a complete national review. The 14 units in the study represent a significant part of the country’s intensive care capacity, but do not include all regions and specialist profiles. Therefore, the generalisation of results across the country should be performed with caution, as regional differences in staff availability and patient severity may be greater than reflected in this study.

Overall, these limitations do not diminish the significance of the study, but emphasise the need for further studies that would complement these results, including full-year, additional clinical indicators, broader hospital coverage and systemic factors affecting intensive care nursing workload. Despite the abovementioned limitations, the study provides substantial evidence-based insight into the actual workload in intensive care units of Latvia and demonstrates the necessity to review and modernise the approaches to personnel planning and care organisation in this field.

## 8. Conclusions

This study provides comprehensive and evidence-based insight into the workload of intensive care nurses in Latvia, revealing consistently high care intensity and significant workload heterogeneity between units, care levels and hospitals. The average time of care was 15.71 h per patient per day, which shows that one nurse is realistically only able to provide care to about one patient. At the same time, the nearly twofold differences between units reveal structural disproportions in access to human resources and organisational models that directly affect both the quality of care and the well-being of staff.

The annual workload dynamics, seasonal fluctuations and structural inertia identified in the ARIMA indicate that intensive care nursing workload is not accidental but predictable and systematically high. Nursing shortages are most closely linked to the amount of care measured by NAS rather than to the type of shift or unit level, which confirms that the shortage problem is directly linked to objective workload rather than administrative settings. Overall, the results indicate that current human resources planning approaches are not sufficient to ensure safe and smooth care.

The study emphasises the need to introduce a uniform NAS-based workload monitoring and personnel planning system in Latvia, which would allow ensuring the quality of care regardless of the institution and reduce the risk of overburdening. At the same time, it is essential to strengthen human resources policy, develop the sustainability of the nursing profession and provide adequate support to staff working in the highest-intensity care environment. These conclusions point to the need for a modernised, data-driven and patient-driven approach to the intensive care organisation in Latvia.

## Figures and Tables

**Table 1:** Descriptive statistics for 14 ICUs.

Indicator	Unit													
	1	2	3	4	5	6	7	8	9	10	11	12	13	14
Number of protocols	871	4604	5164	1031	1744	1597	994	2580	3054	2121	895	911	1127	1386
Median (total)	91.90	54.5	55.50	75.80	83.90	56.50	35.40	56.90	83.25	67.80	91.50	42.60	44.36	77.30
Total Mean (total)	86.41	56.93	58.58	75.79	77.85	65.48	38.77	58.16	81.55	75.21	85.55	54.58	43.37	76.51
Total SD (total)	22.42	17.82	19.57	29.85	27.07	25.46	13.42	21.84	21.11	27.78	30.19	26.72	15.28	27.98
Total (day)	15,284													
Total (night)	12,795													
Total Mean (day)	65.63													
Total SD (day)	26.14													
Total Mean (night)	65.25													
Total SD (night)	25.30													
Min NAS points per 1 nurse	24.80	11.50	11.50	11.60	10.20	11.30	11.50	11.50	15.50	22.60	16.50	19.80	12.60	11.50
Max NAS points per 1 nurse	161.90	136.00	136.50	160.30	153.90	148.70	104.90	154.20	148.60	158.40	141.50	130.90	121.30	176.80
Hours per patient (Mean and SD)	20.74 h (5.38)	13.66 h (4.28)	14.06 h (4.49)	18.19 h (7.16)	18.69 h (6.50)	15.71 h (6.11)	9.30 h (3.22)	13.96 h (5.24)	19.57 h (5.07)	18.05 h (6.66)	20.53 h (7.25)	13.10 h (6.41)	10.65 h (3.67)	18.36 h (6.71)
Shortage rate (%)	56.22	43.47	38.64	22.38	58.32	51.78	14.40	25.62	62.71	4.51	49.78	61.17	62.08	63.34
Workload index	3.12	1.89	1.75	1.79	2.87	2.98	1.66	1.50	3.03	1.52	2.99	3.89	3.07	3.69

**Table 2:** Descriptive statistics at different levels of care (level 1–3).

	1	2	3
Number of protocols	4629	5927	17,523
Number of protocols (day)	2391	3272	9621
Number of protocols (night)	2238	2655	7902
Median (total)	45.60	82.80	60.20
Total Mean (total)	52.46	79.60	64.10
Total SD (total)	23.98	27.97	23.09
Min NAS points per 1 nurse	11.30	10.50	10.50
Max NAS points per 1 nurse	148.70	176.80	158.40
Hours per patient (Mean and SD)	12.59 h (5.76)	19.10 h (6.71)	15.38 h (5.54)
Shortage of nurses (%)	48.08	51.64	38.06
Workload Index	2.90	2.93	1.95

**Table 3:** Descriptive statistics on weekdays, weekends and public holidays.

	Monday	Tuesday	Wednesday	Thursday	Friday	Saturday	Sunday	Public Holiday
Number of protocols	3706	3984	4296	4334	4129	3611	3056	963
Median (total)	59.90	60.75	59.80	60.90	60.40	60.90	62.70	65.20
Total Mean (total)	64.88	65.12	64.25	65.14	65.11	65.57	67.82	69.32
Total SD (total)	25.89	25.26	26.09	25.17	26.25	25.49	26.08	26.73
Min NAS points per 1 nurse	12.80	10.20	11.50	13.80	11.50	11.50	11.50	11.50
Max NAS points per 1 nurse	158.40	151.60	176.80	176.80	161.70	153.90	160.30	176.80
Nursing shortage (%)	43.54	43.18	46.62	45.92	42.43	37.74	61.97	39.35
Workload Index	2.33	2.36	2.42	2.43	2.30	2.19	2.18	2.14

**Table 4:** Nursing workload and staff compliance rates by ICU.

Unit	Number of Patients (Mean)	Number of Nurses (Mean)	Real N/P	Intensity Coefficient	Nurses Required	Compliance (%)
1.	4.07	2	1/0.73	0.038	6.25	43.78
2.	13.98	7.8	1/0.93	0.037	15.16	56.53
3.	15.86	9.6	1/0.92	0.033	17.53	61.36
4.	4.83	2.9	1/1.01	0.026	5.37	77.62
5.	6.07	3	1/0.75	0.045	8.62	41.68
6.	10.00	3.7	1/0.90	0.052	11.93	48.22
7.	5.16	2	1/1.82	0.045	3.33	85.60
8.	8.65	5.7	1/0.98	0.030	8.99	74.38
9.	10.01	5.3	1/0.67	0.040	15.17	37.29
10.	8.53	7.8	1/0.75	0.023	12.72	95.49
11.	3.94	10.4	1/0.74	0.040	5.99	50.22
12.	4.42	1	1/1.38	0.077	3.89	50.21
13.	4.49	1	1/1.56	0.076	3.07	37.92
14.	5.57	2	1/0.82	0.058	7.38	36.66
Level 1	6.52	2.25	1/1.35	0.061	6.34	51.89
Level 2	5.12	2.47	1/0.80	0.043	7.02	48.36
Level 3	12.40	7.77	1/0.87	0.034	14.59	61.94

## Data Availability

The data presented in this study are available upon reasonable request from the corresponding author, as the data utilized in this study are confidential.

## Supplementary Materials

The following supporting information can be downloaded at https://www.mdpi.com/article/10.3390/healthcare14010134/s1. Table S1: Descriptive statistics for 14 ICUs; Table S2: Descriptive statistics at different levels of care (level 1–3); Table S3: Descriptive statistics on weekdays, weekends and public holidays; Figure S1: Boxplot for the number of patients on day and night shifts; Figure S2: Boxplot for NAS values during day and night shifts; Figure S3: Domain heatmap.

## Abbreviations

The following abbreviations are used in this manuscript:

ICU	Intensive Care Unit
NAS	Nursing Activities Score
ANOVA	Analysis of Variance
SD	Standard Deviation
R^2^	Coefficient of Determination
COVID-19	Coronavirus Disease 2019
EU	European Union
N/P	Nurse-to-Patient Ratio
TISS-28	Therapeutic Intervention Scoring System–28
CM	Cabinet of Ministers (Republic of Latvia)
IBM-SPSS	International Business Machines Statistical Package for the Social Sciences
Q1-Q3	First to Third Quartile
IQR	Interquartile Range
Tukey HSD	Tukey Honestly Significant Difference Test
PĒK	Research Ethics Committee (Rīga Stradiņš University)
M	Mean
n	Sample Size
*p*	Probability Value
t	Student’s *t*-Test
CI	Confidence Interval
F	*F*-Statistic
η^2^	Eta Squared (Effect Size)
ω^2^	Omega Squared (Effect Size)
Wald z	Wald *z*-Statistic
ICC	Intraclass Correlation Coefficient
AIC	Akaike Information Criterion
χ^2^	Chi-Square Statistic
β	Standardized Regression Coefficient
ARIMA	Autoregressive Integrated Moving Average
RMSE	Root Mean Square Error
MAE	Mean Absolute Error
MAPE	Mean Absolute Percentage Error
MA	Moving Average
ACF	Autocorrelation Function
PACF	Partial Autocorrelation Function
SOFA	Sequential Organ Failure Assessment
APACHE	Acute Physiology and Chronic Health Evaluation
