# Supplementary material for: A Six-Month Observational Study of Nursing Workload in 14 Latvian Intensive Care Units Using the Nursing Activities Score
Source: Healthcare (Basel). 2026 Jan 5;14(1):134. doi: 10.3390/healthcare14010134 (PMC12786321; doi:10.3390/healthcare14010134)
Supplement: Supplementary file 1 [file healthcare-14-00134-s001.zip › healthcare-4070723-supplementary.pdf]

Supplementary Materials

Table S1. Descriptive statistics for 14 ICUs.

Indicator	Unit													
	**1**	**2**	**3**	**4**	**5**	**6**	**7**	**8**	**9**	**10**	**11**	**12**	**13**	**14**
Number of protocols	871	4604	5164	1031	1744	1597	994	2580	3054	2121	895	911	1127	1386
Number of protocols (day)	470	2518	2698	559	1000	849	489	1458	1824	1123	476	473	580	767
Number of protocols (night)	401	2086	2466	472	744	748	505	1122	1230	998	419	438	547	619
Mean (day)	85.66	57.15	57.78	75.46	76.32	60.01	37.66	59.05	80.45	75.15	87.94	56.78	46.12	75.70
SD (day)	22.76	18.11	19.19	31.23	29.53	25.96	13.31	22.25	22.15	27.31	31.13	28.17	16.41	29.30
Mean (night)	87.29	56.66	59.46	76.19	79.89	64.88	39.84	57.01	83.17	75.27	82.83	52.21	42.51	78.74
SD (night)	22.01	17.46	19.93	28.14	26.21	24.89	13.47	21.28	19.35	28.30	28.89	24.87	13.76	26.09
95% CI Lower	84.92	56.41	58.05	73.97	76.58	64.23	37.93	57.31	80.79	74.03	83.57	52.85	43.47	75.03
95% CI Upper	87.91	57.44	57.69	77.62	79.12	66.73	39.60	59.00	82.29	76.39	87.53	56.32	45.26	77.98
Skewness	-.364	.590	.712	.256	-.501	.694	1.443	.869	.071	.887	-.409	1.127	1.310	.121
Kurtosis	-.752	.748	.350	-.713	-.494	-.201	2.947	1.402	.224	.216	-.897	.290	3.689	.027
Median (total)	91.90	54.5	55.50	75.80	83.90	56.50	35.40	56.90	83.25	67.80	91.50	42.60	44.36	77.30
Total Mean (total)	86.41	56.93	58.58	75.79	77.85	65.48	38.77	58.16	81.55	75.21	85.55	54.58	43.37	76.51
Total SD (total)	22.42	17.82	19.57	29.85	27.07	25.46	13.42	21.84	21.11	27.78	30.19	26.72	15.28	27.98
Total (day)	15284													
Total (night)	12795													
Total Mean (day)	65.63													
Total SD (day)	26.14													
Total Mean (night)	65.25													
Total SD (night)	25.30													
Min NAS points per 1 nurse	24.80	11.50	11.50	11.60	10.20	11.30	11.50	11.50	15.50	22.60	16.50	19.80	12.60	11.50
Max NAS points per 1 nurse	161.90	136.00	136.50	160.30	153.90	148.70	104.90	154.20	148.60	158.40	141.50	130.90	121.30	176.80
Hours per patient (Mean and SD)	20.74 h (5.38)	13.66 h (4.28)	14.06 h (4.49)	18.19 h (7.16)	18.69 h (6.50)	15.71 h (6.11)	9.30 h (3.22)	13.96 h (5.24)	19.57 h (5.07)	18.05 h (6.66)	20.53 h (7.25)	13.10 h (6.41)	10.65 h (3.67)	18.36 h (6.71)
Shortage rate (%)	56.22	43.47	38.64	22.38	58.32	51.78	14.40	25.62	62.71	4.51	49.78	61.17	62.08	63.34

Workload index	3.12	1.89	1.75	1.79	2.87	2.98	1.66	1.50	3.03	1.52	2.99	3.89	3.07	3.69

Table S2. Descriptive statistics at different levels of care (level 1–3).

	1	2	3
Number of protocols	4629	5927	17523
Number of protocols (day)	2391	3272	9621
Number of protocols (night)	2238	2655	7902
Mean (day)	53.56	78.83	63.13
SD (day)	24.89	29.59	23.16
Mean (night)	51.28	80.55	64.06
SD (night)	22.91	25.81	22.99
95% CI Lower	51.77	78.89	63.76
95% CI Upper	53.15	80.31	64.44
Skewness	1.218	-.182	.768
Kurtosis	1.063	-.540	.665
Median (total)	45.60	82.80	60.20
Total Mean (total)	52.46	79.60	64.10
Total SD (total)	23.98	27.97	23.09
Min NAS points per 1 nurse	11.30	10.50	10.50
Max NAS points per 1 nurse	148.70	176.80	158.40
Hours per patient (Mean and SD)	12.59 h (5.76)	19.10 h (6.71)	15.38 h (5.54)
Shortage of nurses (%)	48.08	51.64	38.06
Workload Index	2.90	2.93	1.95

Table S3. Descriptive statistics on weekdays, weekends and public holidays.

	Monday	Tuesday	Wednesday	Thursday	Friday	Saturday	Sunday	Public holiday
Number of protocols	3706	3984	4296	4334	4129	3611	3056	963
Number of protocols (day)	2067	2203	2332	2446	2286	1821	1609	520
Number of protocols (night)	1639	1781	1964	1888	1843	1790	1447	443
Mean (day)	64.14	65.71	63.68	65.15	65.25	66.54	68.71	71.04
SD (day)	25.90	25.89	26.58	25.06	26.94	25.64	26.80	25.76
Mean (night)	65.81	64.38	64.93	65.12	64.94	64.59	66.83	67.29

SD (night)	25.85	24.44	25.48	25.32	25.36	25.30	25.24	25.58
95% CI Lower	64.05	64.33	63.47	64.39	64.31	64.74	66.90	67.69
95% CI Upper	65.72	65.90	65.03	65.89	65.91	66.40	68.75	70.94
Skewness	.582	.484	.594	.648	.624	.626	.546	.560
Kurtosis	-.209	-.329	-.114	.128	.-009	.-083	.-199	.104
Median (total)	59.90	60.75	59.80	60.90	60.40	60.90	62.70	65.20
Total Mean (total)	64.88	65.12	64.25	65.14	65.11	65.57	67.82	69.32
Total SD (total)	25.89	25.26	26.09	25.17	26.25	25.49	26.08	26.73
Min NAS points per 1 nurse	12.80	10.20	11.50	13.80	11.50	11.50	11.50	11.50
Max NAS points per 1 nurse	158.40	151.60	176.80	176.80	161.70	153.90	160.30	176.80
Nursing shortage (%)	43.54	43.18	46.62	45.92	42.43	37.74	61.97	39.35
Workload Index	2.33	2.36	2.42	2.43	2.30	2.19	2.18	2.14

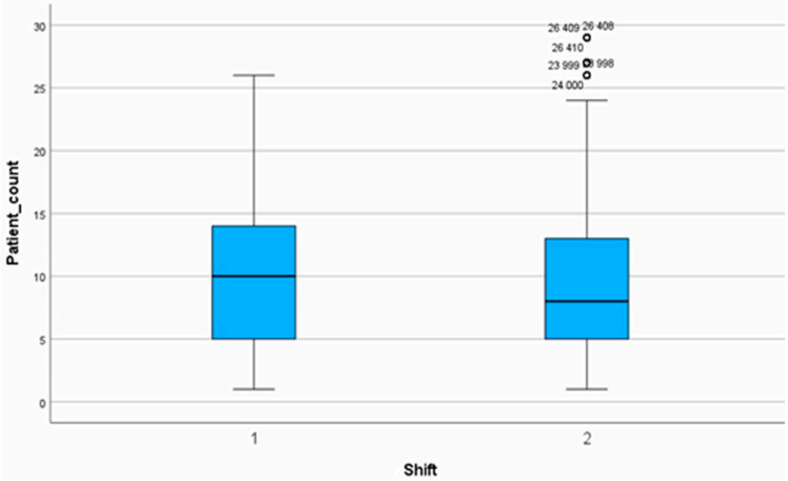

**Shift 1 – Day, Shift 2 - night*

Figure S1. Boxplot for the number of patients on day and night shifts.

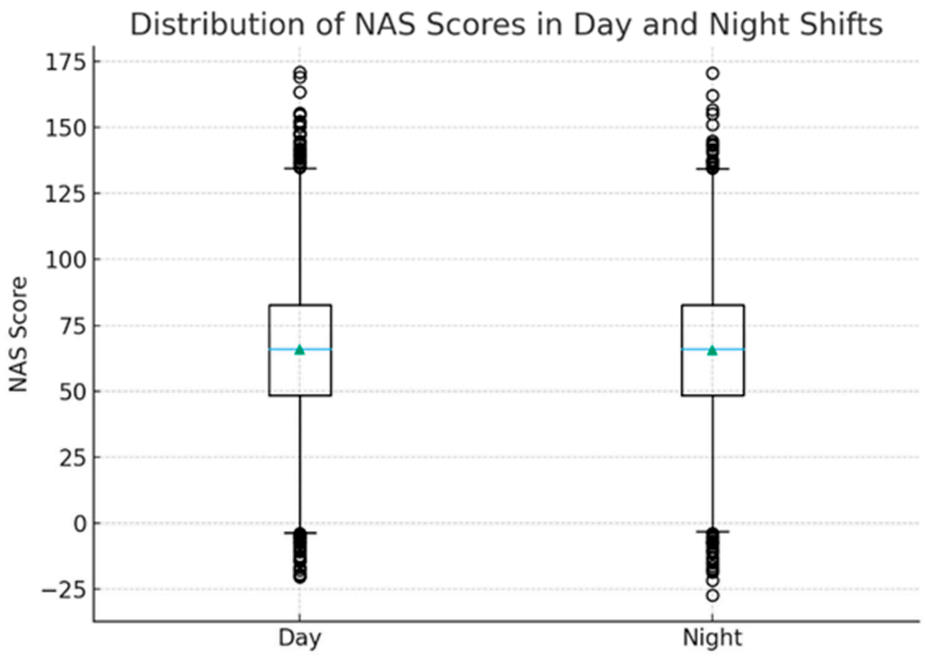

Figure S2. Boxplot for NAS values during day and night shifts.

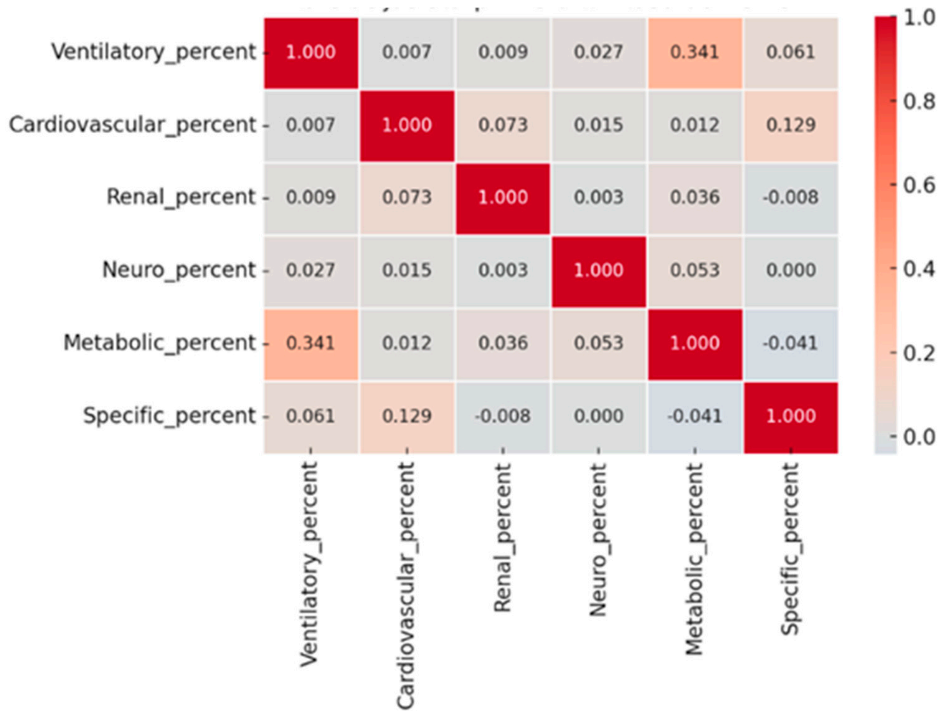

Figure S3. Domain heatmap.
